# NKG2D Ligand Shedding in Response to Stress: Role of ADAM10

**DOI:** 10.3389/fimmu.2020.00447

**Published:** 2020-03-25

**Authors:** Alessandra Zingoni, Elisabetta Vulpis, Luisa Loconte, Angela Santoni

**Affiliations:** ^1^Laboratory Affiliated to Istituto Pasteur Italia-Fondazione Cenci Bolognetti, Department of Molecular Medicine, Sapienza University of Rome, Rome, Italy; ^2^IRCCS Neuromed, Pozzilli, Italy

**Keywords:** NKG2D, NKG2D ligands, shedding, ADAM10, cancer, chemotherapy, senescence

## Abstract

NKG2D is an activating receptor expressed by NK cells and some subsets of T cells and represents a major recognition receptor for detection and elimination of cancer cells. The ligands of NKG2D are stress-induced self-proteins that can be secreted as soluble molecules by protease-mediated cleavage. The release of NKG2D ligands in the extracellular milieu is considered a mode of finely controlling their surface expression levels and represents a relevant immune evasion mechanism employed by cancer cells to elude NKG2D-mediated immune surveillance. A disintegrin and metalloproteinase 10 (ADAM10), a catalytically active member of the ADAM family of proteases, is involved in the cleavage of some NKG2D ligands in various types of cancer cells either in steady state conditions and in response to an ample variety of stress stimuli. Appealing immunotherapeutic strategies devoted to promoting NK cell-mediated recognition and elimination of cancer cells are based on the upregulation of NK cell activating ligands. In particular, activation of DNA damage response (DDR) and the induction of cellular senescence by chemotherapeutic agents are associated with increased expression of NKG2D ligands on cancer cell surface. Herein, we will review advances on the protease-mediated cleavage of NKG2D ligands in response to chemotherapy-induced stress focusing on: (i) the role played by ADAM10 in this process and (ii) the implications of NKG2D ligand shedding in the course of cancer therapy and in senescent cells.

## Introduction

Natural killer cells are innate lymphocytes implicated in the immune surveillance of cancer cells by the integration of signals deriving from numerous cell-surface activating and inhibitory receptors (1). NKG2D is an activating receptor expressed by NK cells, CD8 T lymphocytes, and gdT cells and represents a major recognition receptor able to detect transformed cells. NKG2D ligands are absent or expressed at low levels on healthy cells, and their expression dramatically increases in virus-infected or tumor cells, as well as in response to a number of stress stimuli (2). In the human system, NKG2D recognizes two families of stress-inducible ligands: the MHC class I chain-related protein A/B (MICA/B) and the UL16-binding proteins (ULBP1-6). Multiple levels of regulation govern NKG2D ligand expression including transcriptional, post-transcriptional, and post-translational mechanisms (3, 4). Among post-translational mechanisms, the release of activating ligands in the extracellular milieu through protease-mediated cleavage or by extracellular vesicle secretion is considered a mode of finely controlling surface expression levels of a determined NKG2D ligand. In particular, the shedding of NKG2D ligands by proteases represents an immune evasion mechanism employed by cancer cells since it causes the reduction of NKG2D ligand cell-surface levels, thus rendering cancer cells invisible to NKG2D-mediated surveillance (5–7). It is instead unclear whether the release of NKG2D ligands through extracellular vesicles could result in the reduction of their expression on the cell surface.

The presence of soluble NKG2D ligands in the sera of cancer patients has been extensively documented, and their levels very often correlate with tumor stage and poor prognosis as well as downmodulation of NKG2D on NK and CD8^+^ lymphocytes (5, 6, 8–14). Strategies aimed at removing these soluble ligands from patients’ plasma have been recently proposed (14).

A number of proteases have been involved in the cleavage of NKG2D ligands, including different members of metalloprotease family namely matrix metalloproteases (MMPs) and a disintegrin and metalloproteases (ADAMs) (15–17). Among them ADAM10, a catalytically active member of the ADAM family of proteases, is involved in the cleavage of MICA, MICB, and ULBP-2 molecules in various types of cancer cells. Between all known NKG2D ligands, *MICA* is the most polymorphic non-classical class I gene, with 159 alleles identified to date^1^ (*release December 2019*) (18). Some MICA polymorphisms can significantly affect the shedding process. For instance, the MICA^∗^008 allele is characterized by a short transmembrane domain, and in contrast to other MICA alleles that are sensitive to protease-mediated cleavage, it is bound to the membrane through a glycosylphosphatidylinositol (GPI) anchor (19) and is mainly released associated with exosomes (20). Moreover, the MICA-129 dimorphism, resulting in the valine to methionine substitution at position 129, can affect MICA shedding, but the mechanism behind this is largely unknown (21, 22).

An appealing strategy for immunotherapy devoted to improving NK cell-mediated detection of cancer cells is based on the upregulation of NK cell-activating ligands on the cancer cell surface (23). In this regard, it has been shown that treatment of cancer cells with various chemotherapeutic agents results in upregulation of NKG2D ligands with a consequent enhanced NK cell-mediated recognition and elimination of drug-treated tumor cells (24–30). In particular, activation of DNA damage response (DDR) and the induction of cellular senescence by chemotherapeutic agents are associated with increased expression of NKG2D ligands on cancer cell surface (31, 32). Senescence is a central response to cytotoxic chemotherapies (Chemotherapy-Induced Senescence or CIS) that ends in a state of stable cell cycle arrest (33), and NK cells are involved in the immune surveillance and clearance of senescent cells (34). In the course of therapeutic intervention, modulation of ADAM expression and activity dramatically occurs thus affecting the release of NKG2D ligands in their soluble form. Emerging studies have shown that soluble NKG2D ligands are part of the senescent secretoma known as senescence-associated secretory phenotype (SASP) (35, 36).

Herein, we will discuss about the protease-mediated cleavage of NKG2D ligands on cancer cells in response to stress stimuli focusing our attention on: (i) the role played by ADAM10 in this process; (ii) the mechanisms regulating ADAM10 expression and activity in cancer cells; (iii) the implications of NKG2D ligand shedding in the course of cancer therapy.

## Proteolytic Cleavage of NKG2D Ligands

The extracellular domains of several integral membrane proteins are cut and released as soluble forms from the cell surface by a group of enzymes known as “sheddases” through a process called “ectodomain shedding” (37). The mechanisms regulating NKG2D ligand shedding have been extensively studied, and, as already mentioned, two families of metalloproteases (i.e., MMPs and ADAMs) play a pivotal role in this process. Both MMPs and ADAMs are members of the metzincin subgroup of the zinc protease superfamily which is further divided into serralysins, astacins, matrixins, and adamalysins (38). The matrixins comprise the matrix metalloproteases. Adamalysins are similar to the matrixins in their metalloprotease domains, but contain a unique integrin receptor-binding disintegrin domain. To date, 38 members of the ADAM family have been identified, and in the human system, 13 proteolytically active ADAMs have been characterized.

Among members of ADAM families, roles for ADAM9, ADAM10, and ADAM17 have been demonstrated in the shedding of MICA, MICB, and ULBP-2 molecules. Small interfering RNA-mediated knockdown of ADAM9 resulted in the upregulation of cell-surface MICA expression on hepatocarcinoma cell lines and a concomitant reduction of soluble MICA levels in their culture supernatants (39). However, most of the studies have shown a dominant role of both ADAM10 and ADAM17 in the NKG2D ligand cleavage. Of all the ADAMs, ADAM17 is the most similar to ADAM10 in regard to structure and function. For instance, ADAM10 and ADAM17 are expressed on the cell surface of glioblastoma initiating cells (GIC), and both contribute to an immunosuppressive phenotype by ULBP-2 cleavage. The cell-surface expression of ULBP-2 is enhanced either by selective inhibition of ADAM10 or ADAM17, and following treatment with ADAM10 and ADAM17 specific inhibitors, results in enhanced immune recognition of GIC by NK cells (40). The relative contribution of ADAM10 and ADAM17 in the MICA and MICB-mediated cleavage is still undefined. For example, specific inhibition of ADAM10 or ADAM17 in MICA- or MICB-transduced cells indicate that MICA can be shed by both ADAM10 and ADAM17 (15), while MICB was reported to be selectively shed by ADAM17 (41). However, a pivotal study by Chitdaze et al., highlighted a cell type-specific role of these metalloproteinases in the regulation of MICA/B shedding (42). Indeed, transient RNAi-mediated silencing of ADAM10 or ADAM17, or both, in distinct cancer cell lines revealed a considerable heterogeneity with regards their role in the proteolytic cleavage of MICA and MICB (42). In a model of multiple myeloma, ADAM10 was prevailing with respect to ADAM17 in the MIC-mediated protease cleavage (35). All together, these data strongly suggest that the relative action of ADAM10 versus ADAM17 in MICA/B shedding is a selective feature of a given tumor cell, probably because ADAM activity is regulated at multiple levels which may vary in different tumors. Moreover, the recruitment of both metalloproteases and NKG2D ligands into cholesterol and sphingolipid-enriched microdomains [detergent-resistant membranes (DRMs)] has been suggested to aid efficient proteolytic release of these molecules (41). One common mechanism by which transmembrane proteins are recruited to DRMs relies on modification by fatty acids. In this regard, MICA palmitoylation is a crucial step that determines the recruitment of this ligand to cholesterol-enriched microdomains, thus influencing the shedding process (43). In line with these data, inhibition of fatty acylation and palmitoylation significantly reduced MICB recruitment to DRMs and protease-mediated cleavage (41).

## ADAM10 and Cancer

Several pieces of evidence show that ADAM10 expression and activity is modulated in the course of cancer progression in an ample variety of tumors (44), and usually, increased expression levels of this protease correlate with cancer progression. In this regard, a number of studies have associated ADAM10 with cleavage of the adhesion molecules N-, E-, and VE-cadherins, thus affecting cancer cell adhesion and migration, and β-catenin signaling (45, 46). Notably, ADAM10 is highly expressed in malignant pleural mesothelioma (MPM) and contributes to MPM progression through the generation of N-cadherin fragment that in turn stimulates mesothelioma cell migration (47). Similarly, increased ADAM10 expression is associated with the promotion of the growth of oral squamous cell carcinoma (48) and gastric carcinoma (49). In addition, ADAM10-mediated L1 cell adhesion molecule (LCAM) cleavage is reported to enhance tumor dissemination by increasing cell migration in ovarian and uterine carcinomas (50, 51). Moreover, LCAM is also involved in the motility and invasion of lymphoma, lung carcinoma, and melanoma cells, where ADAM10 seems to be a major L1-sheddase in these tumor cell lines (52, 53). In prostate cancer cells, ADAM10 is highly expressed and contributes to extracellular matrix maintenance and cell invasion (54). ADAM10 is also overexpressed in leukemia (55) and colon cancer (56).

Interestingly, beyond the role of ADAM10 in the NKG2D ligand proteolytic cleavage, this protease has also been described to affect the shedding of other molecules involved in the anti-cancer immune response (57). Eichenauer et al., have shown in human lymphoma that ADAM10-mediated shedding of CD30 determines the failure of antibody-based immunotherapy (58). Recently, it has been reported that ADAM10 also contributes to PDL1 cleavage in breast cancer cells (59). Another intriguing ADAM10 dependent mechanism involved in the immune evasion of tumor cells from NK cell reactivity has been recently described. Of interest, ADAM10 was detected on platelet surface and contributed to NKG2D ligand cleavage from cancer cells (60). Moreover, ADAM10 has been found associated to exosome-like vesicles produced by Hodgkin lymphoma cells; thus, the activity of this protease might be spread in the tumor microenvironment, affecting the shedding process of NKG2D ligands and other substrates (61).

Various stimuli regulate ADAM10 expression and activity including G-protein coupled receptor (GPCR) activators, calcium ionophores and cellular stress (38). In this regard, accumulating evidence indicate that cellular stress produced by a plethora of stimuli, including chemotherapeutic agents, reactive oxygen species (ROS), and ionizing radiations lead to a significant increase of metalloproteinase-mediated shedding of cell-surface molecules, generally related to the enhancement of their expression and/or catalytic activity (35, 62–64).

## Modulation of NKG2D Ligand Shedding in Response to Stress

To date, mechanisms regulating the shedding of NKG2D ligands in response to stress stimuli are not completely known, and although different metalloproteases could be involved in such process, we will focus our attention on a number of studies showing modulation of NKG2D ligand shedding related to a concomitant change of ADAM10 expression/activity.

**FIGURE 1:**
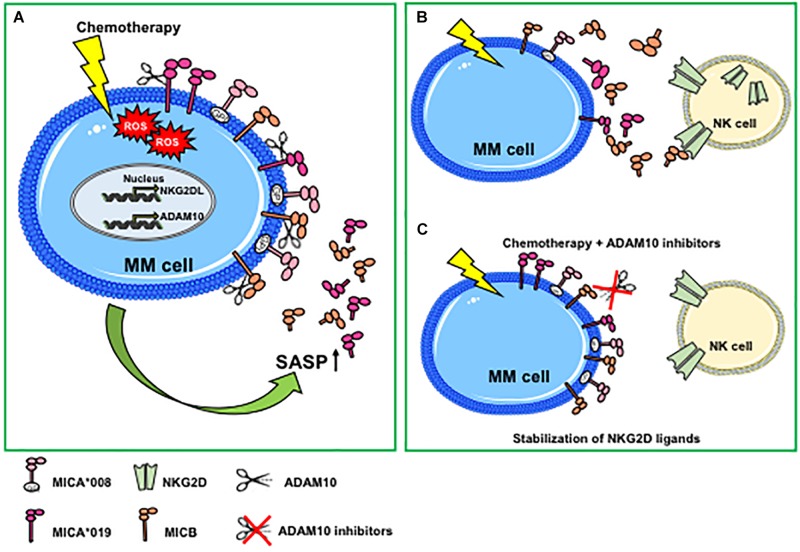
Genotoxic stress promotes ADAM10-mediated shedding of MIC molecules in multiple myeloma. **(A)** Genotoxic stress induced by chemotherapeutic drugs increases cell-surface expression of NKG2D ligands and ADAM10 expression levels and activity through the induction of ROS-dependent DNA damage response. Both NKG2D ligand and ADAM10 upregulation are mainly associated with a senescent phenotype. An increase of the ADAM10-mediated shedding process is observed only for MIC molecules sensitive to protease cleavage (i.e., MICA*019 or MICB), whereas MICA*008 release is not perturbed. **(B)** Genotoxic stress-induced MICA/B shedding favors the accumulation of soluble ligands in the tumor microenvironment that can contribute to the desensitizing of NK cells. **(C)** The combined use of chemotherapeutic agents and ADAM10 inhibitors determines a stabilization of NKG2D ligands on MM cell surface, promoting NK cell-mediated immune surveillance. MM, multiple myeloma; ROS, reactive oxygen species; GPI, glycosylphosphatidylinositol; SASP, senescent-associated secretory phenotype.

In some cellular models, hypoxic conditions may favor the accumulation of soluble NKG2D ligands (65, 66). In this regard, Barsoum and coworkers provided evidence that reduction of nitric oxide levels in prostate cancer cells induced the hypoxia-inducible factor 1α (HIF1α), causing augmentation of ADAM10 expression and a significant increase of MICA secretion in the extracellular milieu (66). A recent work has further shown that ADAM10 upregulation by hypoxia was dependent on the induction of circular_0000977 RNA that served as a sponge to repress miR-153; the authors demonstrated that HIF1A and ADAM10 were direct downstream targets of miR-153 (67). Agents causing DNA damage including genotoxic agents or ionizing radiations promote NKG2D ligand shedding and modulation of ADAM10 expression. For instance, ionizing radiations induced upregulation of ADAM10 and MMP2 proteases, as well as the amount of soluble MICA secreted by lung cancer cells; notably, the combined treatment of ionizing radiation and MMP inhibitors dramatically increased the surface expression levels of MICA, promoting the recognition and killing of cancer cells by NK cells (68). The effects of ionizing radiations on upregulation of ADAM10 expression levels was also shown in a mouse model of breast cancer (69). In line with these studies, etoposide (a topoisomerase II inhibitor able to activate the DDR), enhanced ADAM10 expression (70). In addition, an important role of ADAM10 in the regulation of MICA and MICB shedding in response to the treatment with genotoxic agents was demonstrated in multiple myeloma cell lines and in primary malignant plasma cells derived from patients (35). Of interest, doxorubicin and melphalan increased both ADAM10 expression and activity on multiple myeloma cells and concomitant release of soluble MICA/B. The authors found that ADAM10 upregulation on multiple myeloma cells was dependent on drug-induced production of reactive oxygen species (Figure 1A). It is important to consider that stimulation of MICA shedding by genotoxic drugs was allele-dependent; indeed, the secretion of MICA^∗^008 short allele in its soluble form was not perturbed by pharmacological treatment, whereas MICA^∗^019 long allele was (35) (Figure 1A). By contrast, Kohga et al., have shown that ADAM10 expression was reduced in response to doxorubicin treatment in some hepatocarcinoma cell lines and observed a concomitant decrease of soluble MICA in cell culture supernatants (71). These discrepancies may depend on the different cellular systems and/or experimental conditions used as well as by the MICA allelic variant expressed. In a model of hepatocellular carcinoma, treatment with the histone deacetylase inhibitor, valproic acid (VPA), resulted in increased MICA and MICB cell-surface levels and simultaneous enhancement of their soluble forms in the conditioned media (72). Of interest, combination of VPA and generic protease inhibitors determined a stabilization of cell surface MICA/B on ovarian carcinoma cells (73). Other studies report the inhibition of NKG2D ligand shedding process by chemotherapeutic agents. Treatment of various pancreatic cancer cell lines with the nucleotide analog gemcitabine inhibited ULBP-2 ectodomain shedding through the suppression of ADAM10, thus leading to an enhancement of NK cell cytotoxicity that was strictly dependent on NKG2D/ULBP-2 interaction (74). The authors also found a significant correlation between soluble ULBP-2 serum levels and ADAM10 expression in cancer cells from a cohort of pancreatic ductal adenocarcinoma patients (74). Cell-surface expression levels of MICA significantly increased in response to 5-fluorouracil treatment in hepatocarcinoma and cancer lung cell lines through a mechanism dependent on ADAM10 inhibition (75). Recently, the pharmacological agent disulfiram (DSF) was effective to suppress MICA shedding, impairing ADAM10 activity in hepatocellular carcinoma (76).

A summary of the effects of stress stimuli on both NKG2D ligand shedding and ADAM10 expression/activity in distinct cancer models are reported in Table 1.

**TABLE 1:** Effect of stress conditions on ADAM10 and NKG2D ligand shedding in cancer models.

**Stimulus**	**ADAM10 expression/activity**	**NKG2DL shedding**	**Cancer cell type**	**References**
Hypoxia	↑ Expression	↑ MICA	Prostate	66
Ionizing radiation	↑ Expression	↑ MICA	Lung	68
Ionizing radiation	↑ Expression	n.d.	Breast	69
Doxorubicin melphalan	↑ Expression/activity	↑ MICA*019 MICB	Multiple myeloma	35
Epirubicin	↓ Expression	↓ MICA	Hepatocarcinoma	71
Gemcitabine	↓ Expression	↓ ULBP-2	Pancreatic cancer	74
5-Fluorouracil	↓ Expression	↓ MICA	Hepatocarcinoma lung	75
Disulfiram	↓ Activity	↓ MICA	Hepatocarcinoma	76

## NKG2D Ligands and Senescence: a Double-Edged Sword

Cellular senescence is a cell stress response, leading to cell cycle arrest that is implicated in various physiological processes and in age-related diseases and carcinogenesis (33). Senescent cells can accumulate with age in patients and are found in inflamed and damaged tissues, premalignant lesions, and arrested tumors and after chemo- or radiotherapy. Senescence is considered a tumor-suppressive mechanism, although several studies have also shown that senescent cells can persist and contribute to tumor relapse and to the adverse effects of chemotherapy. DNA-damaging chemotherapies induce tumors to develop a senescence phenotype associated with elevated levels of NKG2D ligands, determining an enhanced recognition and killing by NK cells (31, 32). Senescent cells produce large amounts of soluble factors collectively called SASP. The role of the SASP in tumor progression remains ambiguous and can be detrimental or beneficial, since senescent cells within a tumor can produce secreted factors with both tumor-promoting and tumor-suppressing activities (33, 77). SASP factors include pro-inflammatory cytokines, chemokines, growth factors, and proteases. A large body of evidence describes an increased expression of several metalloproteases, including ADAM10 in cells undergoing senescence, thus contributing to the cleavage and the consequent release of an ample variety of cell-surface molecules in their soluble form. As such, senescence-induced generation of soluble IL-6R was shown to be mediated by ADAM10-dependent ectodomain shedding (78). Zingoni et al., have shown that both MIC release and ADAM10 expression were upregulated in drug-induced multiple myeloma senescent cells, suggesting for the first time that soluble NKG2D ligands are components of tumor cell SASP (35) (Figure 1A). Similarly, a recent study demonstrated that cell culture media of senescent fibroblasts, and epithelial cells contained high levels of soluble MICA. Moreover, the increased release of soluble NKG2D ligands was found also by persistent senescent cells and was associated with expression of different MMPs, thus causing immune evasion by NKG2D-mediated immune detection (36). All together, these observations indicate that soluble NKG2D ligands as components of tumor cell SASP contribute to create a microenvironment suitable for tumor escape. The usage of metalloproteinase inhibitors in combination with chemotherapy can determine the stabilization of NKG2D ligands on the cell surface of drug-induced senescent cancer cells, rendering them more susceptible to NK cell recognition and lysis (35) (Figures 1B,C).

## Conclusion and Perspectives

In past years, cancer therapies designed to kill cancer cells and sustain host anti-tumor immune response represent promising strategies to avoid tumor immune escape. In this context, protease-mediated cleavage of NKG2D ligands represents an immune evasion mechanism that impairs NK cell recognition, since it determines the reduction of NKG2D ligand levels on the cell surface, thus weakening NKG2D-mediated surveillance. In the course of therapeutic intervention, targeting of ADAM10 in conjunction with chemotherapy could be exploited to preserve NKG2D ligands on tumor cell surface, promoting NK cell recognition and killing. In addition, the use of ADAM inhibitors may be an effective therapeutic strategy to restore the immune detection and clearance of persistent, deleterious senescent cells. Another important point of concern relies on MICA polymorphisms. Since MICA shedding is allele-dependent, MICA genotype should be considered to design different personalized strategies aimed at blocking the release of MICA molecules sensitive to protease-mediated cleavage, thus improving NK cell-mediated anti-tumor activity. The development of cancer models, such as transgenic mice overexpressing ADAM species or the generation of selective inhibitors for each ADAM, will be important in determining the role of ADAMs in cancer progression and in the course of chemotherapeutic treatments aimed at potentiating the immune detection of cancer cells.

## Author Contributions

AZ and AS conceived and wrote the manuscript. EV and LL prepared the table and figure and contributed to the writing.

## Conflict of Interest

The authors declare that the research was conducted in the absence of any commercial or financial relationships that could be construed as a potential conflict of interest.

## Footnotes

1 http://www.ebi.ac.uk/imgt/hla/
